# Monocyte Chemotactic Protein-1 (MCP-1) in Patients with Chronic Schistosomiasis Mansoni: Evidences of Subclinical Renal Inflammation

**DOI:** 10.1371/journal.pone.0080421

**Published:** 2013-11-12

**Authors:** Ana Lúcia P. Hanemann, Alexandre B. Libório, Elizabeth F. Daher, Alice Maria C. Martins, Marta Cristhiany C. Pinheiro, Mariana S. Sousa, Fernando Schemelzer M. Bezerra

**Affiliations:** 1 Department of Pathology and Legal Medicine, Federal University of Ceara, Fortaleza, Ceara, Brazil; 2 Department of Clinical Medicine, Federal University of Ceara, Fortaleza, Ceara, Brazil; 3 Department of Clinical Analysis and Toxicology, Federal University of Ceara, Fortaleza, Ceara, Brazil; 4 Department of Community Health, Federal University of Ceara, Fortaleza, Ceara, Brazil; Queensland Institute of Medical Research, Australia

## Abstract

The aim of this study is to investigate renal markers and the biomarker MCP-1 in patients with schistosomiasis mansoni. This is a cross-sectional study with 85 patients aged 5 to 48 years, with a confirmed diagnosis of schistosomiasis mansoni through the Kato-Katz method. The patients were divided in three groups: control (G-I); infected by *S. mansoni* before treatment (G-II) and infected by *S. mansoni* after treatment (G-III). Renal function was evaluated by tubular and glomerular biomarkers and through urinary MCP-1. Patients’ mean age was 23.2±13 years. There was no statistically significant difference between the groups regarding tubular and glomerular function evaluated through the traditional biomarkers. MCP-1 was higher in G-II and G-III, when compared to G-I; p=0.009 and p=0.007, respectively. There was no difference when comparing groups G-II and G-III (p=0.892). Although it was not different among the groups, there was a significant correlation between albuminuria and MCP-1. There was a significant increase in urinary MCP-1 levels in patients with schistosomiasis mansoni, which was associated with albuminuria. This protein has a role in the recruitment of monocytes to injury and inflammation sites . The increase of MCP-1 in the urine evidences that there is silent renal inflammation in these patients and the inflammatory status is not interrupted by specific treatment of the offending agent. Our findings suggest that urinary MCP-1 can be a sensitive marker of renal injury in patients with schistosomiasis mansoni.

## Introduction

The association between infectious endemic diseases and the kidney has already been described [1]. Renal involvement in schistosomiasis is described mainly as glomerular immune damage, clinically presenting as edema, overt proteinuria and hematuria (membranoproliferative glomerulopathy). However, these findings occur only in a few patients with schistosomiasis mansoni, mainly in the hepatosplenic form. It is believed that kidney injury in this disease is secondary to the deposit of circulating immune complex [2]. It is unknown if chronic infection with *Schistosoma mansoni* can lead to slow and progressive renal damage in addition to membranoproliferative glomerulonephritis (MPGN).

Routine markers for renal damage (serum creatinine and albuminuria) lack sensitivity. Even the use of microalbuminuria, one of the most sensitive markers of renal damage currently used in clinical practice is controversial and some studies have suggested that higher normal range values can be related to progressive renal damage [3]. Recently, new biomarkers have appeared and are able to predict future decline in glomerular filtration rate (GFR) [4]. The Monocyte Chemotactic Protein-1 (MCP-1) is one of the recently studied new biomarkers. This protein is expressed in injury and inflammation sites and directs the recruitment of macrophages, which bind to chemokine receptor to promote macrophage adhesion and chemotaxis [5]. The increase in MCP-1 tubular expression can occur in progressive kidney disease and in the presence of interstitial inflammatory infiltrate [6,7]. Urinary MCP-1 has been associated with increased albuminuria in patients with autosomal dominant polycystic kidney disease [8].

To date, there has been no study regarding renal involvement in patients with chronic schistosomiasis mansoni. The aim of this study is to investigate renal markers and the biomarker MCP-1 in patients with schistosomiasis mansoni.

##  Materials and Methods

### Ethical statement

In compliance with the ethical rules governing research on human health provided in Resolution 196/96 of the National Health Council of Brazil, this project was submitted to the Research Ethical Committee of the Department of Physiology and Pharmacology of Universidade Federal do Ceara and approved under n. 165/09. Before selection, all families attended a lecture detailing the objectives and procedures of this study. Those who chose to participate had the study explained in full at home. Informed written consent was obtained from the study participants or, in the case of minors, from their parents or guardians.

### Patient selection and diagnosis

This is a cross-sectional study carried out in Planalto do Cajueiro, a district in the town of Maranguape, Ceara State, Northeast of Brazil. This district is a small community distributed across five urban blocks, according to a survey carried out by the town hall. The area is surrounded by two streams and *Biomphalaria straminea* is the intermediate host of schistosomiasis mansoni. Patients older than 60 and younger than 2 were excluded, as well as those with diabetes mellitus, hypertension, systemic lupus erythematosus, rheumatoid arthritis, chronic kidney disease and any other condition that could cause kidney injury. Moreover, a urine microscopy study was performed to exclude significant leukocyturia (higher than 5 white blood cells per high-power field). A total of 85 individuals were selected, according their status for *S. mansoni* infection. Group I (GI): non-infected subjects, demonstrated by negative serology through ELISA and Kato-Katz methods - n=24; Group II (GII); infected subjects with no previous treatment (n=30) and group III (G III) infected subjects treated between 6-12 months before study inclusion. In the last group, reinfection was excluded by performing new Kato-Katz test (3 samples) at study inclusion. 

### Study design

Blood samples were collected to perform sorting of individuals by ELISA using soluble adult worm antigen (SWAP) of *S. mansoni*. After this analysis, feces were collected for Kato-Katz testing [9], to determine the aforementioned groups. New blood and urine samples were collected to perform kidney function evaluation through the estimation of Glomerular Filtration Rate (eGFR), serum and urine electrolytes (calcium, magnesium, phosphorus, sodium and potassium), urinalysis, albuminuria and urinary MCP-1.

### Biochemical analysis

Serum creatinine and urinary albumin were determined by the kinetic-colorimetric method and the results were expressed in mg/dL. After the sample analysis, the eGFR was calculated by the Cockcroft-Gault [10] and Schwartz formulas [11]. Electrolytes were measured using Labtest^®^ kits and the results were used to estimate fractional excretion. Urinary MCP-1 measurement was performed through ELISA using the R&D Systems^®^ kit, Inc (Minneapolis, MN, USA). The MCP-1 value was corrected by urinary creatinine.

### Treatment

All patients who were positive for the presence of *S. mansoni* eggs in the stool were treated with Praziquantel^®^ at a dose of 60 mg/kg of body weight in a single dose. The drug was dispensed under medical supervision at the community’s public health center. Patients in the GIII had their treatment efficacy and no reinfection confirmed by a new Kato-Katz test.

### Statistical analysis

Data were expressed as mean ± standard deviation. Statistical analysis was carried out using the SPSS program, version 19.0 (SPSS Inc, Chicago, IL, USA). After testing for normality using the K-S test, differences between the means of multiple variables were inferred trough One-Way ANOVA test and Bonferroni post-test and the correlations were evaluated according to Pearson’s test. The established confidence interval was 95%, and “p” values <0.05 were considered statistically significant.

## Results

Patients’ mean age was 23.2 ± 13 years. There was no significant difference regarding age (p=0.50) and gender (p=0.18) among the groups.

The cut-off value used for ELISA was 0.283 (±2SD). The mean reading of ELISA in groups I, II, III (the latter, before treatment) were, respectively, 0.18, 0.48 and 0.62. None of ELISA tests were performed after treatment.

In the infected group (GII), there were *S. mansoni* eggs in all cases and 3 cases also had other parasitic diseases, while in the post-treatment group (GIII), only 1 patient had other parasitic infection, with *Trichuris trichiura* being the other parasite found.

There was no difference regarding tubular function among the three groups (Table 1). Regarding glomerular function, there was also no difference in GFR and albuminuria among the groups (Table 1). The urinalysis showed no abnormal results.

**Table 1:** Renal function biomarkers in patients with schistosomiasis mansoni and controls.

**Biomarker**	**Non-infected G-I (n=24)**	**Infected G-II (n= 30)**	**Post-treatment G-III (n= 31)**	***p value***
**Age (years)**	21,58 ± 14,02	22,07 ± 14,07	25,42 ± 13,41	n.s.
**U _pH_**	5.94 ± 0.43	5.91± 0.44	6.02 ± 0.51	n.s.
**FE_Ca_^+^ (%)**	1.15 ± 0.63	1.35 ± 2.89	1.43 ± 0.98	n.s.
**FE_Mg_ (%)**	5.44 ± 6.30	3.72 ± 4.07	3.51 ± 4.55	n.s.
**FE_Pi_ (%)**	15.66 ± 11.84	16.94 ± 18.72	14.91 ± 12.86	n.s.
**FE_Na_^+^ (%)**	0.73 ± 0.51	0.82 ± 0.29	0.83 ± 0.48	n.s.
**FE_K_^+^ (%)**	3.04 ± 1.71	3.54 ± 2.86	4.98± 5.02	n.s.
**eGFR (mL/min/1.73m^2^)**	121.94 ± 28.44	124.35 ± 26.26	113.21 ± 18.16	n.s.
**Albuminuria (mg/dL)**	7.20 ± 7.69	5.41 ± 5.31	5.34 ± 5.31	n.s.

Data expressed as Mean±Standard deviation. Quantitative variables were compared by One-Way ANOVA and Bonferroni post-test.

U: urinary; FE: fractional excretion; MCP-1: monocyte chemotactic protein-1; eGFR: estimated glomerular filtration rate; n.s.: non-significant.

**Figure 1:**
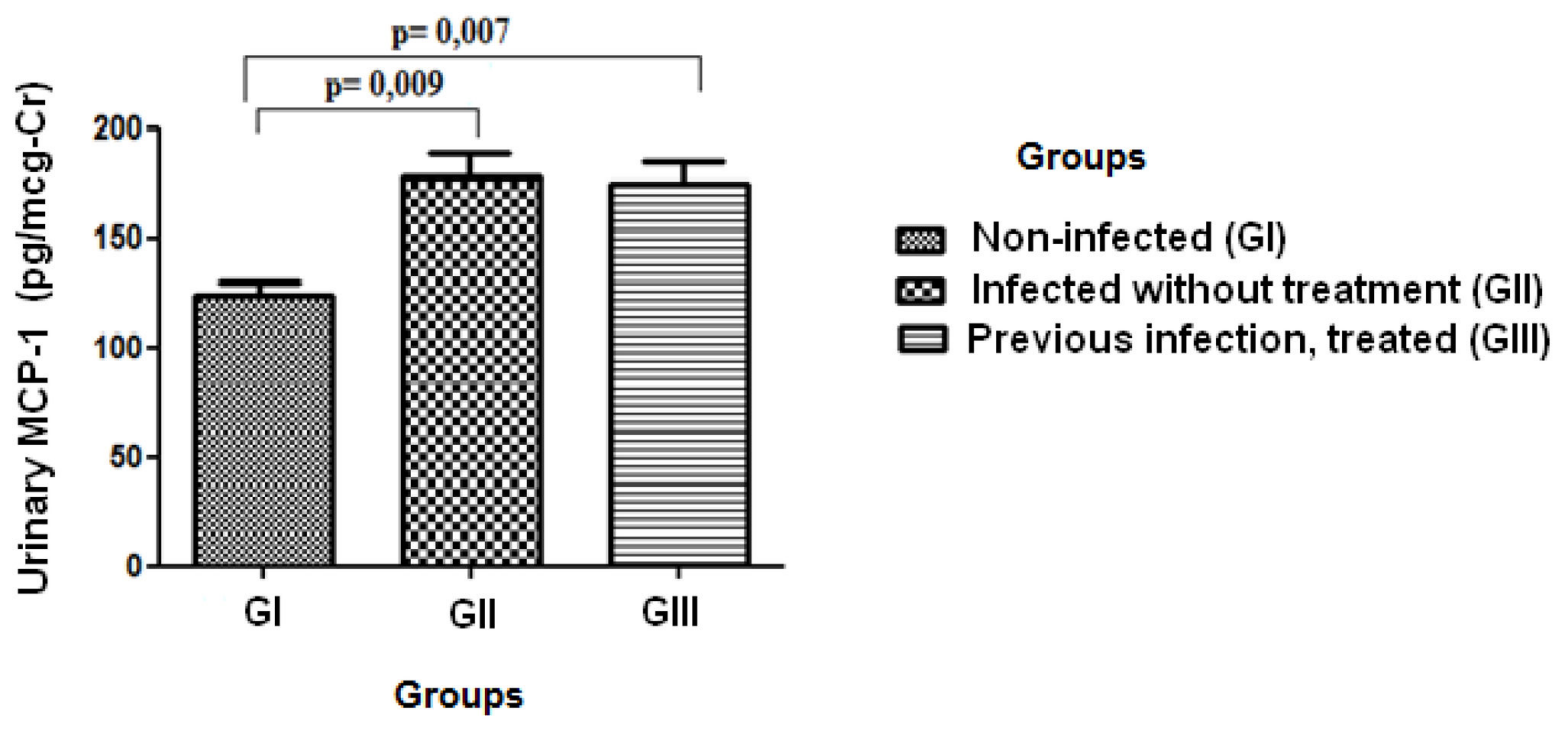
Urinary MCP-1 levels in the studied groups. The study was carried out in a small community distributed across five urban blocks, in Planalto do Cajueiro in Maranguape city, Ceara State, Northeast of Brazil. This is a low endemic area for *S. mansoni*. All the three groups were submitted to MCP-1 urinary test.

**Figure 2:**
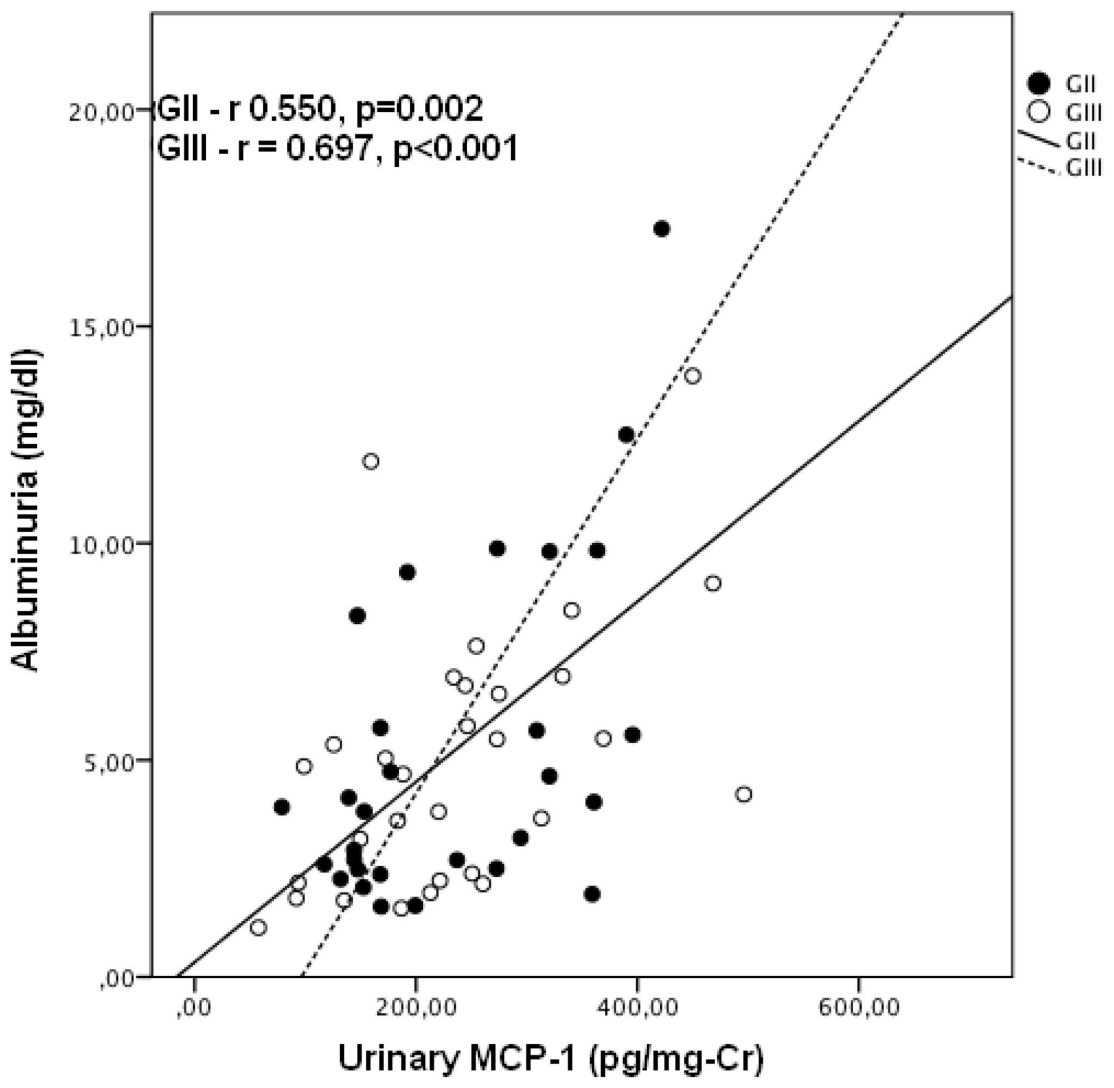
Correlation between urinary MCP-1 and albuminuria. The study was carried out in a small community distributed across five urban blocks, in Planalto do Cajueiro in Maranguape city, Ceara State, Northeast of Brazil. This is a low endemic area for *S. mansoni*. There was a significant correlation between traditional biomarkers and MCP-1. GII- Infected without treatment; GIII- Previous infection, treated.

MCP-1 levels were significantly higher in GII (178 ± 97pg/mg-Cr) and GIII (175 ± 87pg/mg-Cr), when compared to GI (123 ± 48pg/mg-Cr), p=0.009 and p=0.007, respectively, but there was no difference when comparing GII and GIII (p=0.892) (Figure 1). Although albuminuria was within the normal range in all except one patient, and there were no differences among the groups, there was a significant correlation between MCP-1 and albuminuria in patients with active or previous infection r=0.463, p=0.01. This association was present in both, active or previous infection – Figure 2. There was no significant correlation between any tubular function parameter and urinary MCP-1 (table 2).

**Table 2:** Correlation between urinary MCP-1 and tubular function parameters.

**Parameter**	**Correlation**	**p**
**U _pH_**	-0.065	0.619
**FE_Ca_^+^ (%)**	-0.120	0.382
**FE_Mg_ (%)**	-0.111	0.421
**FE_Pi_ (%)**	0.077	0.575
**FE_Na_^+^ (%)**	-0.089	0.526
**FE_K_^+^ (%)**	-0.057	0.682

The correlations were evaluated according to Pearson’s test.

 Although the intensity of the humoral response to the parasite can cause some damage by the immune complexes deposition, we found no correlation among IgG-ELISA and the markers used as MCP-1 (r = 0.031, p = 0.810), albuminuria (r=-0.070, p=0.612) and GFR (r=-0.129, p=0.322).

## Discussion

For the first time, we have shown that schistosomiasis mansoni infection can induce a chronic renal inflammatory status, demonstrated by an increment in urinary MCP-1. However, this inflammatory status is not interrupted by the specific treatment of the offending agent.

Most patients in the present study were young with low probability of underlying kidney disease. Although there was no difference among the three groups in relation to urinary albumin excretion rate, an increase in urinary MCP-1 was observed in patients with active or treated schistosomiasis mansoni infection. This finding suggests that urinary MCP-1 may be a more sensitive marker of renal damage than urinary albumin excretion rate.

In other renal diseases, urinary MCP-1 has been correlated with urinary albumin excretion rate, glomerular filtration rate reduction and other features of renal injury. To date, studies have included metabolic (diabetes mellitus), immunologic (lupus nephritis) and genetic (autosomal dominant polycystic kidney disease) kidney disorders [12,13]. To the best of our knowledge, this is the first study to disclose an infectious disease that promotes an increase in urinary MCP-1. If this finding is limited to schistosomiasis mansoni or may be initiated by any chronic infectious state is unknown, but it is possible to speculate that any chronic inflammatory state can induce urinary MCP-1.

Another marker of glomerular kidney injury that has been studied is cystatin C. It is an endogenous marker of renal dysfunction; in fact, it is a more adequate marker of GFR, rather than a primary AKI biomarker. Furthermore, there are also tubular biomarkers such as Kidney Injury Molecule-1(KIM-1), Neutrophil gelatinase-associated lipocalin (NGAL), Interleukin-18 and N-acetyl-β-D-glucosaminidase (NAG). KIM-1and NGAL have been studied in humans and rodents. KIM-1 is not detectable in normal kidney tissue or urine, but is expressed at very high levels in kidney tubular epithelial cells of rodents and humans after ischemic injury and NGAL is expressed by neutrophils and epithelial cells including those of the proximal tubule [14].

In a recent study, kidney injury was characterized by high levels of proteinuria, Kidney Injury Molecule-1 (KIM-1) and urea, in guinea pigs infected and non-infected with *Trypanosoma cruzi* Y strain [15].

It is noteworthy the fact that even 6 months after schistosomiasis mansoni treatment, there was no reduction in urinary MCP-1. Although most patients had albumin excretion rate within the normal range, a positive and significant correlation was observed between albuminuria and urinary MCP-1. It is already know that even patients with high-normal albumin excretion rate are at high-risk of developing chronic renal failure. So, this study suggest that young patient with schistosomiasis mansoni infection are at higher risk to develop clinically significant kidney injury than elder patients.

These data strongly suggests that there was a chronic and asymptomatic inflammation in these patients infected with *S. mansoni* and this correlated with albuminuria levels, which were within normal limits. As schistosomiasis mansoni is a disease that predominates in youngerindividuals, these patients are at higher risk of renal function loss and should benefit from measures to slow kidney disease progression. MCP-1 seems to be a useful early biomarker for schistosomal nephropathy.
